# Association between History of Vaccination and Symptoms at Diagnosis of Coronavirus Disease 2019

**DOI:** 10.31662/jmaj.2023-0079

**Published:** 2023-09-27

**Authors:** Toshihiko Shiraiwa, Mitsuyoshi Takahara, Yoshifumi Maeno, Kaoru Yamamoto, Yuka Shiraiwa, Yoko Yoshida, Norio Nishioka, Kotomi Kurihara, Yuko Yamada

**Affiliations:** 1 Shiraiwa Medical Clinic, Kashiwara, Japan; 2 Department of Diabetes Care Medicine, Osaka University Graduate School of Medicine, Suita, Japan

**Keywords:** Coronavirus disease 2019, Symptom at diagnosis, History of vaccination, Body temperature

## Abstract

**Introduction:**

We investigated the association between history of vaccination for coronavirus disease 2019 (COVID-19) and symptoms at its diagnosis.

**Methods:**

We retrospectively analyzed 2566 consecutive individuals suspected of having COVID-19 and visited a designated clinic between January and September 2022 (1733 were diagnosed with COVID-19, and 816 tested negative for COVID-19) in Japan. The individuals were divided by vaccination history for COVID-19.

**Results:**

In the COVID-19-free individuals, the vaccination was not significantly associated with any symptoms. Contrarily, those with COVID-19 demonstrated an inverse relationship between the vaccination and body temperature; the adjusted mean value was higher by 0.01°C, 0.04°C, 0.09°C, 0.27°C, and 0.34°C and 0.48°C in individuals vaccinated 2-4, 4-6, 6-8, 8-10, and >10 months before and those unvaccinated, respectively, than in those vaccinated within 2 months (*P* = 0.96, 0.41, 0.081, 0.006, 0.004, and <0.001). Furthermore, among the affected population, individuals vaccinated long before or never vaccinated more frequently complained of fatigue and headache; the adjusted odds ratios of those vaccinated >10 months before and those unvaccinated compared with those vaccinated within 2 months were 2.53 and 2.45 for fatigue and 2.53 and 2.17 for headache (all *P* < 0.05). Contrarily, the prevalence of rhinorrhea, sore throat, and cough was higher in recently vaccinated individuals (adjusted odds ratios of those vaccinated within 2 months versus those unvaccinated, 2.40, 2.46, and 2.46; all *P* < 0.05).

**Conclusions:**

Symptoms at the COVID-19 diagnosis differed with the vaccination history. Information on vaccination history would be worth using when suspecting COVID-19 based on symptoms.

## Introduction

The coronavirus disease 2019 (COVID-19) pandemic has significantly affected people’s daily lives. At present, the most effective strategy for COVID-19 prevention is vaccination. Vaccines against COVID-19 have been developed and become clinically available since December 2020 ^(1)^. In Japan, vaccination was started exclusively among healthcare personnel in February 2021 and the elderly in April 2021, followed by people with comorbidities and workers at facilities for the elderly; subsequently, it was expanded to all citizens ^(2)^. Growing evidence indicates that vaccination significantly reduces the probability of getting infected with COVID-19 and becoming clinically ill once infected ^(3)^. However, it remains unclear whether vaccination would modify symptoms at COVID-19 onset. COVID-19 is typically accompanied by fever, fatigue, and upper respiratory symptoms including rhinorrhea and sore throat. These relevant symptoms strongly indicate the possibility of COVID-19. Japan has downgraded the categorization of COVID-19 in May 2023, and more practitioners have been involved in its diagnosis. If previous vaccination modifies symptoms at COVID-19 onset, it would affect the application of practical strategies for suspecting COVID-19 in the framework of primary care, as well as public health.

The present study aimed to determine whether clinical manifestations would vary with the time since the last COVID-19 vaccination in individuals diagnosed with COVID-19 in Japan.

## Materials and Methods

We retrospectively analyzed 2566 consecutive individuals suspected of having COVID-19 who visited Shiraiwa Medical Clinic, Kashiwara City, Osaka, Japan, between January and September 2022. The clinic is a medical facility designated by the local government to provide primary care to patients with COVID-19. The study period covers the time of the sixth and seventh waves of the COVID-19 pandemic in Japan. The omicron variant was an overwhelmingly dominant strain during the study period in Japan.

Of the 2566 individuals suspected of having COVID-19, 1733 tested positive, whereas the remaining 833 individuals tested negative. These 833 individuals were used as controls to determine whether the history of COVID-19 vaccination would be unrelated to the symptoms in the COVID-19-free population. Of the 1733 individuals with COVID-19, 1435 (82.8%) were diagnosed via laboratory testing (either a nucleic acid amplification test or an antigen test), whereas the remaining 298 individuals (17.2%) were diagnosed based only on the government’s mandate to diagnose close-contact individuals who develop relevant symptoms as COVID-19-positive without laboratory testing when the pandemic overwhelms local medical facilities and exceeds the testing capacity. The present study also determined whether the association between vaccination and symptoms would be affected by these diagnostic means (clinical versus laboratory diagnosis).

The individuals’ relevant information, including sex, age, symptoms (body temperature and presence of fatigue, headache, rhinorrhea, sore throat, and cough), history of vaccination, and risk factors for severe illness, were retrospectively collected from their medical records. The individuals were divided into those with and without a history of COVID-19 vaccination. Those with a history of vaccination were further classified according to the months after the last vaccination (<2, 2-4, 4-6, 6-8, 8-10, and >10 months). According to the domestic clinical guideline, the risk factors for severe illness included malignant neoplasm, chronic respiratory disease, chronic kidney disease, diabetes mellitus, hypertension, dyslipidemia, cardiovascular disease, cerebrovascular disease, obesity (body mass index ≥30 kg/m^2^), smoking, immunosuppressive drug use, late pregnancy, and human immunodeficiency virus infection ^(4)^.

This study was conducted in accordance with the Declaration of Helsinki and was approved by the Institutional Review Board of Osaka University Hospital (approval number, 22315(G1); approval date, October 26, 2022). The requirement to obtain any informed consent was waived.

### Statistical analyses

Data were expressed as mean ± standard deviations for continuous variables and as percentages for categorical variables, unless otherwise indicated. A two-sided *P* value of <0.05 was considered statistically significant, and 95% confidence intervals were reported where appropriate. The association between vaccination history and body temperature was investigated using linear regression models, whereas the association between vaccination history and other symptoms (presence of fatigue, headache, rhinorrhea, sore throat, and cough) was explored using logistic regression models. All models included history of vaccination in addition to age, sex, and number of risk factors for severe illness. The adjusted mean body temperature and proportions of other symptoms by vaccination status were estimated based on these regression models. In addition, interaction analysis was conducted to determine whether the association between the history of vaccination and symptoms would be affected by the diagnostic means (clinical versus laboratory diagnosis). Missing data were addressed via multiple imputation using the chained equation process. In the procedure, five imputed datasets were generated, and the analytic results were combined according to Rubin’s rules. All statistical analyses were conducted using R version 3.6.0 (R Development Core Team, Vienna, Austria).

## Results

The mean age of the study population was 42 ± 20 years, and 48.2% of them were men. Their clinical characteristics are summarized in Table 1.

**Table 1.** Clinical Characteristics of the Study Population.

	Overall population (n = 2566)	People with COVID-19 (n = 1733)	People without COVID-19 (n = 833)
Male sex	1237 (48.2%)	825 (47.6%)	412 (49.5%)
Age (years)	42 ± 20	41 ± 19	44 ± 21
Risk factors for severe illness			
No risk factor	931 (45.5%)	663 (50.5%)	268 (36.5%)
1 risk factor	647 (31.6%)	410 (31.2%)	237 (32.3%)
2 risk factors	263 (12.8%)	151 (11.5%)	112 (15.3%)
3 risk factors	112 (5.5%)	45 (3.4%)	67 (9.1%)
4 risk factors	59 (2.9%)	27 (2.1%)	32 (4.4%)
≥5 risk factors	36 (1.8%)	18 (1.4%)	18 (2.5%)
History of vaccination for COVID-19			
Unvaccinated	377 (15.7%)	299 (18.4%)	78 (10.1%)
Vaccinated	2023 (84.3%)	1330 (81.6%)	693 (89.9%)
Number of vaccinations			
Once	13 (0.6%)	9 (0.7%)	4 (0.6%)
Twice	1105 (54.6%)	697 (52.4%)	408 (58.9%)
Three times	823 (40.7%)	567 (42.6%)	256 (36.9%)
Four times	82 (4.1%)	57 (4.3%)	25 (3.6%)
Months after the last vaccination
<2 months	227 (12.1%)	109 (8.8%)	118 (18.3%)
2-4 months	279 (14.8%)	183 (14.8%)	96 (14.9%)
4-6 months	609 (32.4%)	412 (33.4%)	197 (30.6%)
6-8 months	394 (21.0%)	252 (20.4%)	142 (22.0%)
8-10 months	167 (8.9%)	116 (9.4%)	51 (7.9%)
>10 months	203 (10.8%)	163 (13.2%)	40 (6.2%)

Data are expressed as mean ± standard deviation or frequency (percentage). Data were missing on risk factors for severe illness in 518 individuals (20.2%), on history of vaccination for COVID-19 in 166 (6.5%), and on months after the last vaccination in 144 (7.1% of the 2023 vaccinated individuals).

### History of vaccination and symptoms

**Figure 1.**
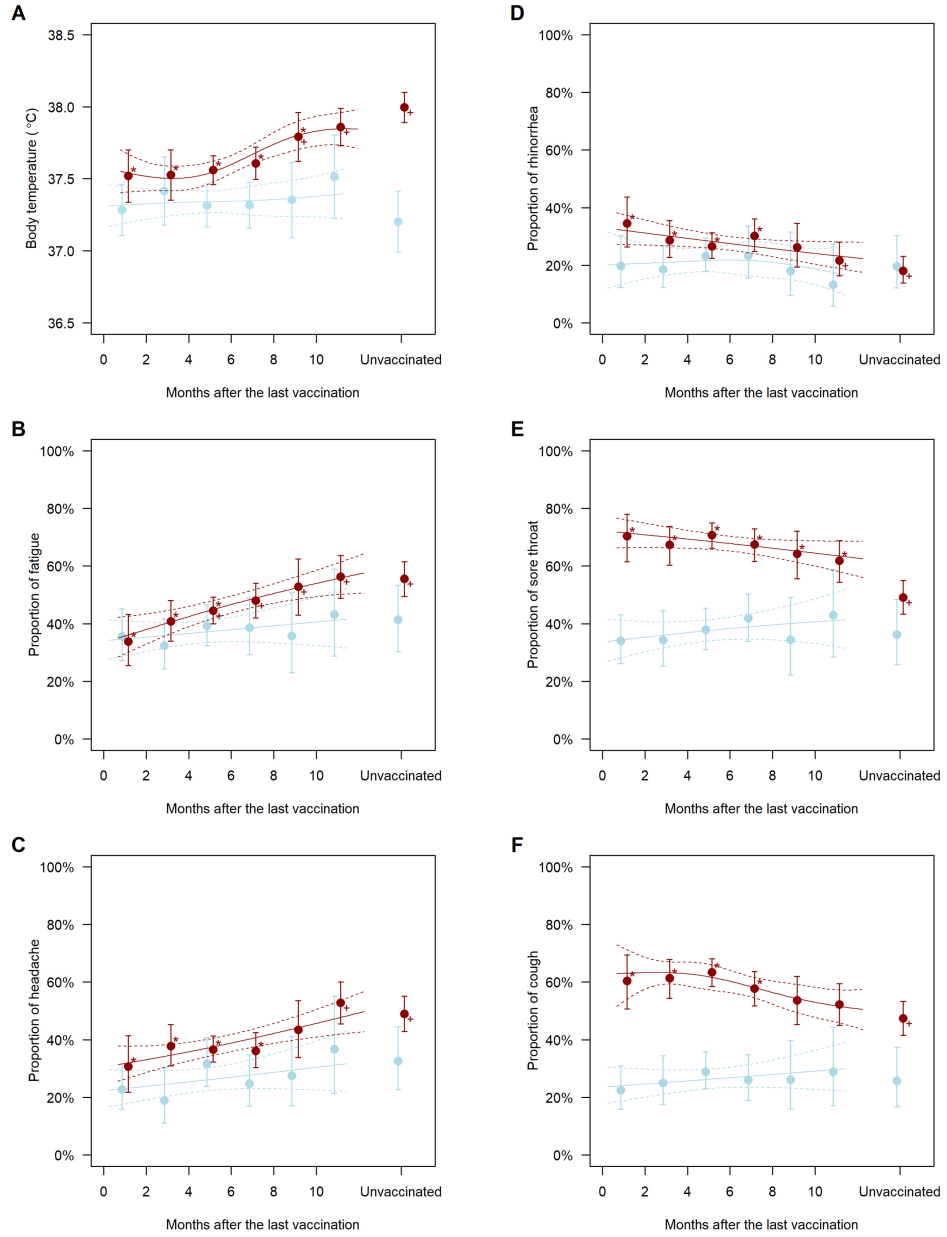
Symptoms by history of vaccination in individuals without (blue plots) and with (red plots) COVID-19. Data are the mean body temperature (A) and the proportions for fatigue (B), headache (C), rhinorrhea (D), sore throat (E), and cough (F) adjusted for sex, age, and risk factors for severe illness. The error bars indicate 95% confidence intervals. ^*^P < 0.05 versus unvaccinated individuals; ^+^*P* < 0.05 versus those vaccinated within 2 months. The solid line indicates the cubic spline for months after the last vaccination, whereas the dotted lines denote the upper and lower limits of its 95% confidence interval.

In the COVID-19-free population, the symptoms did not significantly vary with the time since the last COVID-19 vaccination (blue plots in Figure 1A, 1B, 1C, 1D, 1E and 1F). Contrarily, the symptoms significantly varied with the time since the last vaccination in the COVID-19 population (red plots in Figure 1A, 1B, 1C, 1D, 1E and 1F). Individuals vaccinated long before or never vaccinated had a higher body temperature than those recently vaccinated (Figure 1A). Furthermore, the adjusted mean body temperature of those vaccinated 2-4, 4-6, 6-8, 8-10, and >10 months before and those unvaccinated was higher by 0.01°C (95% confidence interval, −0.29-0.30; *P* = 0.96), 0.04°C (−0.16-0.24; *P* = 0.68), 0.09°C (−0.12-0.30; *P* = 0.40), 0.27°C (0.02-0.53; *P* = 0.037), and 0.34°C (0.13-0.55; *P* = 0.002) and 0.48°C (0.26-0.70; *P* < 0.001), respectively, than that of those vaccinated within 2 months. Furthermore, individuals vaccinated long before or never vaccinated had a higher prevalence of fatigue and headache (Figure 1B and C); the adjusted odds ratios of those vaccinated >10 months before and those never vaccinated versus those vaccinated within 2 months were 2.53 (1.50-4.24; *P* = 0.001) and 2.45 (1.51-3.96; *P* < 0.001) for fatigue and 2.53 (1.46-4.37; *P* = 0.001) and 2.17 (1.32-3.56; *P* = 0.002) for headache, respectively. Contrarily, the prevalence of rhinorrhea, sore throat, and cough was higher in recently vaccinated individuals (Figure 1D, 1E and 1F); the adjusted odds ratios of those vaccinated within 2 and 2-4 months before versus those never vaccinated were 2.40 (1.45-3.97; *P* = 0.001) and 1.83 (1.19-2.82; *P* = 0.006) for rhinorrhea, 2.46 (1.52-3.96; *P* < 0.001) and 2.14 (1.45-3.14; *P* < 0.001) for sore throat, and 2.46 (1.52-3.96; *P* < 0.001) and 2.14 (1.45-3.14; *P* < 0.001) for cough, respectively. The estimated differences in body temperature and odds ratios of symptoms relative to those vaccinated within 2 months and those never vaccinated are presented in Table 2.

**Table 2.** Association between History of Vaccination and Symptoms in People Diagnosed with COVID-19.

	Body temperature (°C)	Fatigue	Headache	Rhinorrhea	Sore throat	Cough
versus <2 months before						
2-4 months before	0.01 [−0.29-0.30] (P = 0.96)	1.34 [0.84-2.16] (P = 0.22)	1.37 [0.76-2.48] (P = 0.29)	0.76 [0.47-1.25] (P = 0.28)	0.87 [0.52-1.46] (P = 0.60)	1.04 [0.63-1.71] (P = 0.88)
4-6 months before	0.04 [−0.16-0.24] (P = 0.68)	1.57 [1.01-2.43] (P = 0.044)	1.31 [0.79-2.17] (P = 0.30)	0.69 [0.44-1.07] (P = 0.096)	1.02 [0.65-1.60] (P = 0.93)	1.14 [0.72-1.80] (P = 0.58)
6-8 months before	0.09 [−0.12-0.30] (P = 0.40)	1.81 [1.15-2.84] (P = 0.010)	1.28 [0.76-2.15] (P = 0.35)	0.82 [0.51-1.32] (P = 0.42)	0.88 [0.55-1.39] (P = 0.57)	0.90 [0.56-1.44] (P = 0.65)
8-10 months before	0.27 [0.02-0.53] (P = 0.037)	2.19 [1.30-3.71] (P = 0.003)	1.73 [0.88-3.41] (P = 0.11)	0.68 [0.38-1.20] (P = 0.18)	0.76 [0.44-1.30] (P = 0.31)	0.76 [0.45-1.29] (P = 0.31)
>10 months before	0.34 [0.13-0.55] (P = 0.002)	2.53 [1.50-4.24] (P = 0.001)	2.53 [1.46-4.37] (P = 0.001)	0.52 [0.31-0.89] (P = 0.018)	0.68 [0.42-1.12] (P = 0.13)	0.72 [0.44-1.18] (P = 0.19)
Unvaccinated	0.48 [0.26-0.70] (P < 0.001)	2.45 [1.51-3.96] (P < 0.001)	2.17 [1.32-3.56] (P = 0.002)	0.42 [0.25-0.69] (P = 0.001)	0.41 [0.25-0.66] (P < 0.001)	0.59 [0.37-0.94] (P = 0.027)
versus Unvaccinated						
<2 months before	−0.48 [−0.70-−0.26] (P < 0.001)	0.41 [0.25-0.66] (P < 0.001)	0.46 [0.28-0.76] (P = 0.002)	2.40 [1.45-3.97] (P = 0.001)	2.46 [1.52-3.96] (P < 0.001)	1.70 [1.06-2.71] (P = 0.027)
2-4 months before	−0.47 [−0.65-−0.28] (P < 0.001)	0.55 [0.37-0.81] (P = 0.003)	0.63 [0.42-0.96] (P = 0.030)	1.83 [1.19-2.82] (P = 0.006)	2.14 [1.45-3.14] (P < 0.001)	1.76 [1.22-2.54] (P = 0.002)
4-6 months before	−0.44 [−0.58-−0.29] (P < 0.001)	0.64 [0.47-0.87] (P = 0.005)	0.60 [0.44-0.83] (P = 0.002)	1.65 [1.12-2.43] (P = 0.011)	2.51 [1.81-3.46] (P < 0.001)	1.93 [1.38-2.68] (P < 0.001)
6-8 months before	−0.39 [−0.54-−0.23] (P < 0.001)	0.74 [0.52-1.06] (P = 0.096)	0.59 [0.41-0.84] (P = 0.004)	1.97 [1.28-3.02] (P = 0.002)	2.15 [1.50-3.09] (P < 0.001)	1.52 [1.07-2.16] (P = 0.020)
8-10 months before	−0.20 [−0.40-−0.01] (P = 0.043)	0.90 [0.56-1.43] (P = 0.64)	0.80 [0.49-1.32] (P = 0.37)	1.62 [0.98-2.67] (P = 0.057)	1.86 [1.21-2.85] (P = 0.005)	1.29 [0.85-1.95] (P = 0.23)
>10 months before	−0.14 [−0.30-0.03] (P = 0.11)	1.03 [0.70-1.53] (P = 0.87)	1.17 [0.80-1.70] (P = 0.42)	1.26 [0.79-2.00] (P = 0.34)	1.68 [1.13-2.49] (P = 0.010)	1.22 [0.84-1.75] (P = 0.29)

Data are adjusted estimates (regression coefficients for body temperature and odds ratios for other symptoms) [95% confidence intervals] (*p* values). All estimates were adjusted for sex, age, and risk factors for severe illness.

### Laboratory versus clinical diagnosis

**Figure 2.**
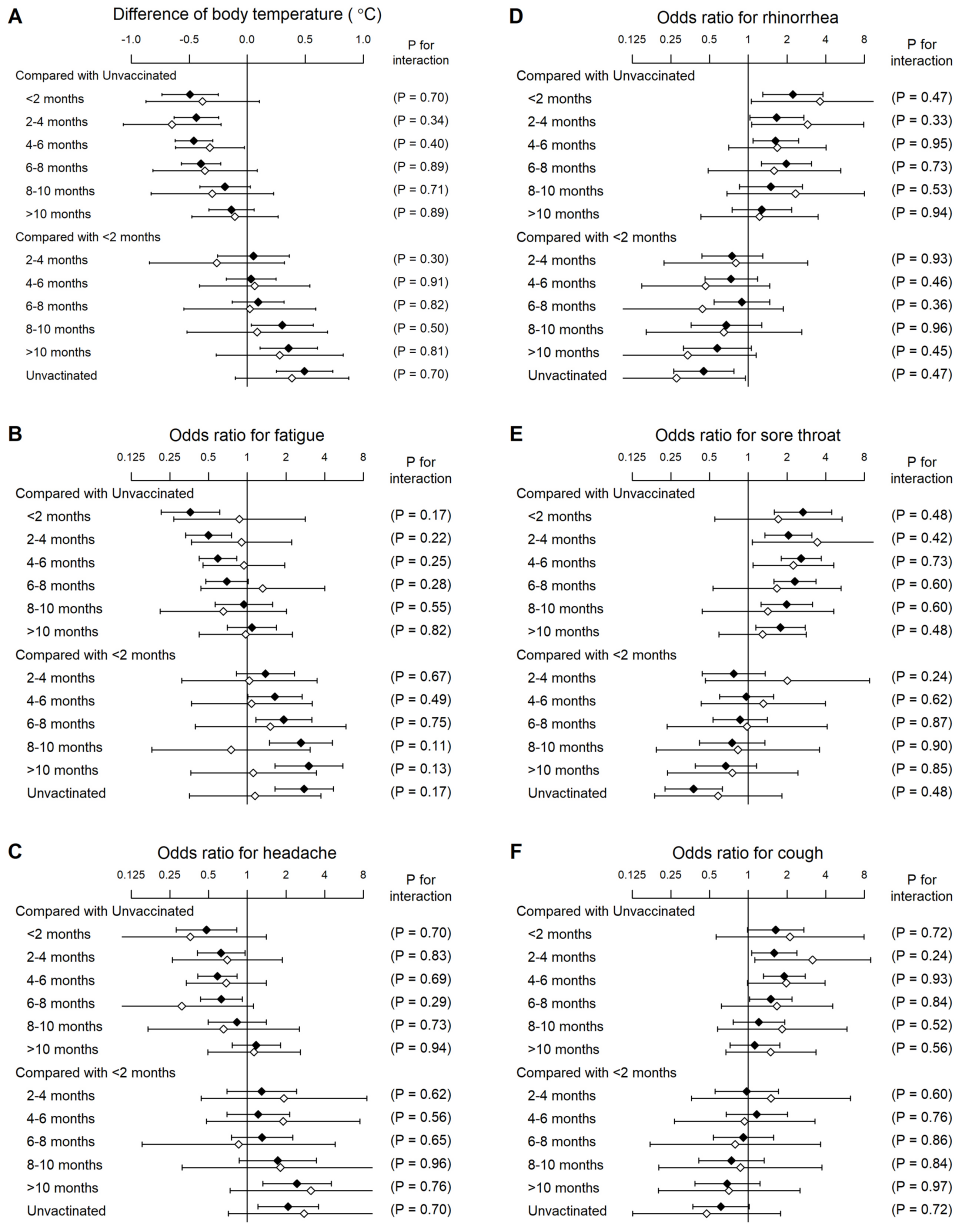
Interaction effect of clinical (blank diamonds) versus laboratory (filled diamonds) diagnosis with COVID-19 on the association between history of vaccination and symptoms in COVID-19 individuals. Data are the mean body temperature (A) and the proportions for fatigue (B), headache (C), rhinorrhea (D), sore throat (E), and cough (F) adjusted for sex, age, and risk factors for severe illness. The error bars indicate 95% confidence intervals. *P* values are for the interaction of clinical and laboratory diagnoses with COVID-19.

In the COVID-19 population, 298 (17.2%) were clinically diagnosed without laboratory testing. Interaction analysis revealed that the associations between vaccination and symptoms did not significantly differ between laboratory and clinical diagnoses (Figure 2).

## Discussion

This study showed that vaccination history significantly affected symptoms at the COVID-19 diagnosis. Individuals vaccinated long before or never vaccinated had a higher body temperature and more frequently complained of fatigue and headache than those recently vaccinated. Contrarily, the prevalence of rhinorrhea, sore throat, and cough was higher in recently vaccinated individuals. Suspicion of COVID-19 based on symptoms is the first and crucial step to detect the infection. The present findings would serve for practical screening strategies in the framework of primary care and public health.

In the COVID-19-free individuals, vaccination was reasonably unrelated to symptoms. However, it was associated with symptoms in individuals diagnosed with COVID-19, indicating that vaccination would alter the manifestation of relevant symptoms at the COVID-19 diagnosis. The symptoms that were more prevalent in individuals vaccinated long before or never vaccinated were fever, fatigue, and headache. Fever is generally associated with severe viral infections, particularly emerging viral infections where the virus is novel to the host ^(5)^. Its key trigger is pyretic cytokines released from local macrophages and other leucocytes involved in the innate immune system ^(6)^. The released cytokines give a signal to the temperature control center of the hypothalamus to increase the thermal set point, resulting in fever ^(5), (6)^. These cytokines also trigger headache ^(7)^, often accompanied by fatigue ^(5)^; however, the detailed mechanisms remain unknown. Although data on cytokine profiles were unavailable in this study, pyretic cytokines, including tumor necrosis factor alpha and interleukin (IL)-6, have been reported to increase in individuals with COVID-19 ^(8), (9)^. In individuals vaccinated long before or never vaccinated, the adaptive immune reaction was assumed to be insufficient. Therefore, their immune response to SARS-CoV-2 infection would be largely dependent on their innate immune system. This might be the reason why they were more likely to experience fever, fatigue, and headache related to macrophage-derived cytokines.

Unlike fever, fatigue, and other systemic symptoms induced by macrophage-released cytokines, local respiratory symptoms, including rhinorrhea, sore throat, and cough, are induced by local responses in the infected airway via inflammatory mediators such as bradykinin and prostaglandins ^(10), (11)^. The inflammatory mediator response is independent of innate immune cells and is likely to be observed regardless of vaccination. Vaccination would reduce susceptibility to fever but not to these respiratory symptoms. In recently vaccinated individuals, local respiratory symptoms would be dominant over fever and other systemic symptoms. On the other hand, the inflammatory mediator response might have a slower onset than the pyretic cytokine response, and local respiratory symptoms might be preceded by fever during viral infection ^(5)^. Individuals vaccinated long before or never vaccinated might visit the clinic and be diagnosed with COVID-19 based on the symptom of fever, before subsequent local respiratory symptoms appear. This might be the reason why recently vaccinated individuals more frequently complained of local respiratory symptoms at the time of diagnosis than those vaccinated long before or never vaccinated. However, the sequential order of symptom appearance during SARS-CoV-2 infection is yet to reach the general consensus; it rather significantly varies from patient to patient ^(12), (13)^. Another possible explanation for the more frequent local respiratory symptoms in vaccinated individuals is the involvement of adaptive immunity. Vaccination has been reported to increase adaptive immune responses, including SARS-CoV-2-specific IgG levels, during COVID-19 infection ^(14), (15)^. Mucus production and airway response to bradykinin are known to be increased by adaptive immunity, particularly by the Th2 cell response ^(16), (17)^. Vaccinated individuals have been reported to have higher eosinophil count during COVID-19 infection than unvaccinated ones ^(18)^, suggesting that vaccination enhances not only the Th1 cell response but also the Th2 cell response during the infection. This partial activation might increase the probability of experiencing local respiratory symptoms during SARS-CoV-2 infection in recently vaccinated individuals. However, there is no direct evidence showing that increased adaptive immunity modifies or affects local respiratory symptoms. The mechanism remains a matter of speculation.

This study had several limitations. First, only symptoms at diagnosis were analyzed; their time course and the symptoms that emerged after diagnosis remain unknown. Second, the present study was just an epidemiological study, not a pathophysiology investigation, and the mechanism between vaccination history and symptoms is also still unknown. Third, blood tests were seldom conducted at the COVID-19 diagnosis, and there was little information on surrogate markers of inflammatory response. C-reactive protein (CRP) levels were measured in only 41 out of 1733 individuals with COVID-19, and ferritin levels were not measured in any of them. Statistical analysis revealed that there was no significant association between vaccination history and CRP levels (*P* = 0.47, tested using one-way analysis of variance for log-transformed CRP levels by vaccination history). However, this result would be considerably affected by our insufficient sample size; we were unable to deny that there was no association between vaccination history and CRP levels. Fourth, detailed data on comorbidities, including their severity, treatments, and relevant laboratory examinations, were unavailable. Information on the viral variant, type of vaccine, cytokine profiles during the COVID-19 infection, and immune responses after vaccination, such as SARS-CoV-2-specific IgG levels, was also unavailable. Fifth, the present study analyzed individuals suspected of having COVID-19 at a single center. Although thousands of cases were included in this study, some selection biases might affect the findings. Future studies are warranted to validate the current findings.

In conclusion, history of vaccination significantly affected symptoms at the COVID-19 diagnosis. Individuals vaccinated long before or never vaccinated had a higher body temperature and more frequently complained of fatigue and headache than those recently vaccinated. Contrarily, the prevalence of rhinorrhea, sore throat, and cough was higher in recently vaccinated individuals.

## Article Information

### Conflicts of Interest

None

### Acknowledgement

The authors would like to thank Megumi Shindo, Yuko Kusuda, Toshiko Ozaki, Yuko Fujimoto, Yuriko Taniwa, Yoshie Ohtani, Sawa Inoue, Hiroko Sakamoto, and Aya Takano, nurses of Shiraiwa Medical Clinic; Hiroko Konishi, Arisa Kawano, Ayako Yamashita, Eri Kobayashi, Mitsuhiro Asai, Yuki Minobe, Takayuki Minooka, and Sachiko Yumiki, biomedical laboratory scientists of Shiraiwa Medical Clinic; Aiwa Osada, Yukari Morioka, Kaoru Shibata, Chiaki Kai, Misaki Ohara, Ibuki Matsumoto, Natsuho Kira, Tomomi Miko, Ayame Nakajima, Haruhi Hirose, Atsuko Shimamura, Ayami Mizoguchi, Natsuki Aono, Megumi Watanabe, and Yuka Hara, registered dietitians of Shiraiwa Medical Clinic; Taku Oishi, Rika Kugita, Mika Yamaguchi, Mayumi Yamamoto, Tomoko Ichikawa, Miki Ohno, Ayumi Matsuda, Kaori Yamanaka, Natsuko Ohue, Noriko Mitsui, Nanami Tsuchino, and Hiroyo Hase, medical assistants of Shiraiwa Medical Clinic; and Naoko Ogawa, Mayumi Yamamoto, Shingo Yun, Hiromi Yasuda, Toshiaki Ujino, Yasuko Takenaka, Akiko Ohashi, and Chikako Maeda, pharmacists of Smile Pharmacy, for their great help in providing primary care to patients suspected of having COVID-19.

### Author Contributions

Toshihiko Shiraiwa wrote the manuscript. Mitsuyoshi Takahara analyzed the data and wrote the manuscript. Yoshifumi Maeno, Kaoru Yamamoto, Yuka Shiraiwa, Yoko Yoshida, Norio Nishioka, Kotomi Kurihara, and Yuko Yamada contributed to the discussion and reviewed/edited the manuscript. All authors read and approved the final manuscript.

### Approval by Institutional Review Board (IRB)

22315(G1), by the Institutional Review Boards of Osaka University Hospital
